# The Potential of Agglomerated Cork for Sandwich Structures: A Systematic Investigation of Physical, Thermal, and Mechanical Properties

**DOI:** 10.3390/polym11122118

**Published:** 2019-12-17

**Authors:** Claudia Sergi, Jacopo Tirillò, Fabrizio Sarasini, Enrique Barbero Pozuelo, Sonia Sanchez Saez, Christoph Burgstaller

**Affiliations:** 1 Department of Chemical Engineering Materials Environment, Sapienza-Università di Roma and UdR INSTM, Via Eudossiana 18, 00184 Roma, Italy; fabrizio.sarasini@uniroma1.it; 2 Department of Continuum Mechanics and Structural Analysis, University Carlos III of Madrid, Avda de la Universidad 30, Leganés, 28911 Madrid, Spain; ebarbero@ing.uc3m.es (E.B.P.); ssanchez@ing.uc3m.es (S.S.S.); 3 Transfercenter für Kunststofftechnik GmbH, Franz-Fritsch-Straße 11, A-4600 Wels, Austria; christoph.burgstaller@tckt.at

**Keywords:** agglomerated cork, PVC foam, bio-based core, eco-friendly, morphological characterization, thermal characterization, TGA, DMA, thermal conductivity, wettability

## Abstract

Considering the major role played by sandwich structures in many fields where high stiffness-to-weight ratio is required, the selection of a suitable core material is of paramount importance. In order to face the environmental problems related to waste disposal, the selection of an eco-friendly core material is now included in the design criteria of sandwich structures. Agglomerated cork is recognized as a good solution that combines satisfactory mechanical performances and eco-sustainability. Many research studies individually addressed cork’s morphological, thermal, and mechanical features without providing a comprehensive overview of the relationships that exist between them. In this work, the investigation of the peculiar cork morphology allowed learning more about its good insulation capacity and its impressive recovery capability. The use of dynamic mechanical analysis (DMA) and thermogravimetric analysis (TGA) clarified the influence of temperature on both flexural and compressive performances. The effect of testing parameters such as temperature and speed on agglomerated cork properties was validated through statistical analysis. Moreover, to highlight agglomerated cork advantages and drawbacks, the work provides also a comparison with more traditional polyvinylchloride (PVC) foams commonly used in industrial applications.

## 1. Introduction

Sandwich structures are gaining greater importance in many fields such as transportation and buildings, fulfilling structural and semi-structural requirements. They combine high strength, stiffness, and lightness ensuring high stiffness-to-weight ratio and good structural performances. The selection of the right core material is a key point in the production of sandwiches to optimize mechanical performances and weight of the structure.

Core materials are traditionally produced employing synthetic materials and can be grouped into four main categories: foams, honeycombs, web core, and corrugated core [1]. Polyurethane (PUR), phenolic, polystyrene (PS), and polyvinylchloride (PVC) are the traditional polymers used to obtain core foams, whereas polypropylene (PP), Nomex, and aluminum are usually employed to produce honeycomb structures. Despite the good results obtained with these core materials, the environmental problems related to pollution and the more restrictive regulations in the field of waste disposal make it necessary to find an alternative to these synthetic materials.

A bio-based core material from renewable resources would allow reducing the carbon footprint in the production phase, the emission of greenhouse gases, and the consumption of energy and chemicals, as already happens with the replacement of common glass fibers with basalt and vegetable ones [2]. It would also ensure a total or partial biodegradation of the sandwich structure at the end of its life cycle. Many research studies were carried out to investigate balsa wood mechanical properties in order to assess its feasibility as a core material. The majority of these studies focused on its flexural performances, investigating residual bending properties [3], flexural fatigue behavior [4], and flexural creep behavior [5]. Another eco-friendly solution as a core material was recognized in agglomerated cork that, thanks to its cellular structure, displays high acoustic and thermal insulation properties, good damping capabilities, and excellent dimensional recovery. Other important characteristics of cork include a good fire resistance, a low permeability to gas and liquid, and a high chemical stability [6,7]. The use of agglomerated cork also allows to take advantage of the waste generated in wine stopper production that otherwise would be lost, thus maximizing the usage of the harvested cork.

Cork is the suberous bark of the *Quercus suber* L. that grows in areas characterized by low rainfall, high humidity, and a good exposition to sunlight, such as western Mediterranean areas, i.e., Portugal, Spain, Italy, and North Africa. Cork annual production is around 201 thousand tons with Europe producing more than 80% [7,8,9,10]. Cork chemical composition contributes significantly to its peculiar properties such as high elasticity and low permeability, but it is strongly dependent on plant geographic origin, climate conditions, tree dimensions, and age [7]. Suberin is a glyceridic polyester made up of glycerol and long-chain fatty acids, and it is the main cork cell-wall component representing approximately 58 wt. % [6]. Its ribbon-like structure is responsible for cork’s elastic properties, allowing cell wall bending and collapse [8,11]. Lignin is an aromatic cross-linked polymer and is the second most important component representing 26 wt. %. Lignin provides cork with its structural rigidity and compression resistance [8]. Cork displays an anisotropic cellular structure that causes anisotropy in its properties [7], an issue that can be solved using agglomerated cork. The random orientation of the granules in agglomerated cork plates tends to counteract their anisotropy, leading to a material with fairly isotropic properties.

Many studies addressed the compression performances of cork and agglomerated cork taking into account parameters such as density, porosity, and test speed [12,13]. Other studies focused on the characterization of cork surface behavior [14] and cork thermal properties [15]. The aim of this work is to provide, in a single paper, a comprehensive investigation of agglomerated cork properties including a morphological, thermal, and mechanical characterization, with a view to disclosing the relationships that exist between its characteristics and properties, as a function of temperature and density. Morphological characterization allowed identifying the particular closed-cell structure of cork and justifying, in this way, its remarkable recovery capabilities and good insulation properties. The use of thermal analyses, such as thermogravimetric analysis (TGA) and dynamic mechanical analysis (DMA), provided additional information to understand the evolution of agglomerated cork flexural and compressive properties with temperature. Furthermore, statistical analysis permitted disclosing the influence of parameters such as temperature and density on agglomerated cork’s mechanical properties. In order to shed light on agglomerated cork’s advantages and drawbacks, the work also includes a comparison with more traditional PVC foams commercially used as core materials.

## 2. Materials and Methods

### 2.1. Materials

Three agglomerated corks obtained by the same cork type but with different densities were used in this work in order to investigate the influence of this parameter on cork properties. The PVC foams selected had the same three density values in order to provide a valid comparison of the results obtained. Agglomerated corks with average densities of 140 kg/m^3^ (NL10), 200 kg/m^3^ (NL20), and 250 kg/m^3^ (NL25) were provided by Amorim Cork Composites. NL10 has a grain size of 2/4 mm, whereas NL20 and NL25 have grain sizes of 0.5/2 mm. Cork granules are bonded together through a polyurethane binder specifically formulated for cork in order to be compatible with all the resins used in the composites industries. PVC foams with an average density of 130 kg/m^3^ (Divinycell HP130), 200 kg/m^3^ (Divinycell HP200), and 250 kg/m^3^ (Divinycell HP250) were provided by Diab. All core materials were supplied as plates with 15 mm of thickness.

### 2.2. Morphological Characterization and Surface Wettability

Morphological characterization of agglomerated corks and PVC foams was carried out through a field-emission scanning electron microscope (FE-SEM) MIRA3 by Tescan (Brno, Czech Republic). Before the observation, all specimens were sputter-coated with a thin layer of gold in order to prevent charging.

Sample surface wettability was investigated by measuring the contact angles of water droplets. Tests were performed at room temperature using an optical analyzer (OCA 15Pro, DataPhysics Instruments GmbH, Filderstadt, Germany) and selecting a static sessile method with a droplet volume of 3 µL. The testing liquid used was Milli-Q ultrapure water characterized by 18 MΩ∙cm. Contact angle values were determined by analyzing droplet shape through the DataPhysics SCA 20 software module. At least 10 contact angles were measured for each material in different areas of the sample.

### 2.3. Thermal Characterization

**Figure 1:**
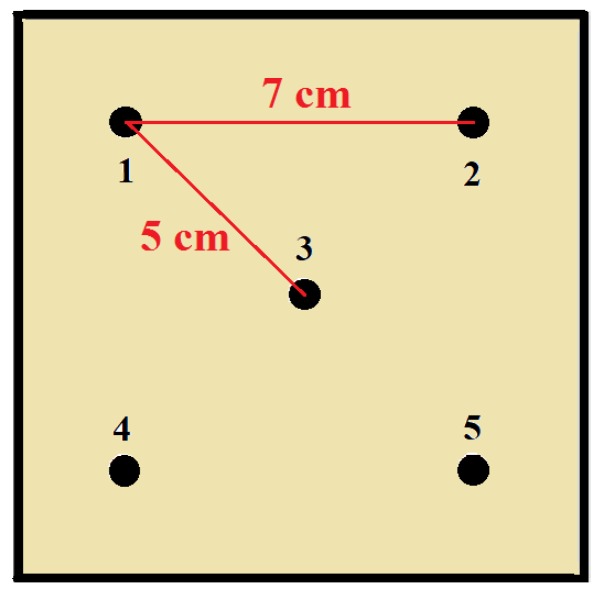
Schematic representation of specimen sampling points for thermal conductivity analysis.

The thermal conductivity, thermal diffusivity, specific heat, and resistance value (*R*-Value) of agglomerated corks and PVC foams were determined through a TCi thermal conductivity analyzer by C-Therm (C-Therm Technologies Ltd., Fredericton, Canada), which is a modified transient plane source. The spiral heating element of the sensor is heated with a known amount of electric current. This causes an increase in temperature at the interface between the sensor and the sample, which in turn induces a change in sensor voltage drop. The rate of increase in sensor voltage is used to determine thermal conductivity, which is inversely proportional to this rate. Thermal conductivity is directly measured by the instrumentation, whereas thermal diffusivity, specific heat, and *R*-value are calculated by the software using the actual density sample value. Tests were carried out on five different points of a squared sample of 100 mm length and 15 mm thickness, as schematically depicted in Figure 1. Ten measurements were performed for each point. Untreated samples and conditioned samples were tested in order to investigate moisture effect on material thermal properties.

The thermal stability of agglomerated corks and PVC foams was investigated through thermogravimetric analysis (TGA) performed with a Setsys Evolution by Setaram (Caluire, France). Samples were heated with a heating rate of 10 °C/min in a nitrogen atmosphere in order to avoid oxidative reactions. Sample mass changes were recorded as a function of temperature.

Variations of core materials mechanical performances with temperature were also investigated through dynamic mechanical analysis (DMA). Tests were performed with a rheometer Anton Paar MCR 501 (Rivoli, Italy) by submitting samples to a heating rate of 2 °C/min in torsion with a frequency of 1 Hz. A gaseous nitrogen flow of 160 mL/min was used to keep a controlled atmosphere during cooling and heating steps. Storage and loss moduli trends with temperature were studied, and glass transition temperature was evaluated.

### 2.4. Mechanical Characterization

A quasi-static characterization of the samples was carried out in shear, bending, and compression. Shear test parameters were selected in agreement with ASTM C273. Tests were performed in tension loading with a speed of 2 mm/min on samples with 300 mm length, 50 mm width, and 15 mm thickness. The shear stress was applied to the sample gluing it to the loading plates through a structural epoxy resin, Elan-tech ADH 46.46. Shear tests on NL10 and NL20 were performed on a Zwick/Roell Z010 (Ulm, Germany) universal testing machine with a loading cell of 10 kN, whereas NL25 was tested with a Instron 5584 (Pianezza, Italy) with a loading cell of 150 kN because of its higher mechanical performance.

Material flexural performances were evaluated through a three-point bending test that was carried out according to ASTM C393 and ASTM D7250 on samples with 250 mm length, 50 mm width, and 15 mm thickness. A support span of 150 mm and a test speed of 6 mm/min were used. Tests were performed at room temperature, 40 °C, 60 °C, and 80 °C in order to investigate the influence of temperature on agglomerated cork and PVC flexural performances. The selected temperature range was dictated by the need to analyze the progression of agglomerated cork behavior at different temperatures up to an upper limit at which the mechanical properties of polyurethane binders normally experience a strong reduction [16]. Samples were conditioned for two hours before testing in order to achieve a homogeneous temperature across the whole specimen.

Compression tests were performed on cubic samples with a 15 mm edge as a function of both temperature and test speed in order to investigate the effect of these parameters on agglomerated cork and PVC compression properties. With respect to the temperature effect, it was evaluated by carrying out tests with a 5 mm/min speed at room temperature, 40 °C, 60 °C, and 80 °C, always on pre-conditioned samples. Material strain rate sensitivity at room temperature was investigated with tests performed at 5 mm/min, 25 mm/min, 50 mm/min, 100 mm/min, 150 mm/min, and 200 mm/min. Material recovery capability as a function of strain rate was investigated by submitting all the samples tested at room temperature to a periodic measurement. Both flexural and compression tests were performed on a Zwick/Roell Z010 universal testing machine equipped with a climatic chamber.

### 2.5. Statistical Analysis of Data

Flexural and compression tests were supported by statistical analysis. Inferential statistics were used to determine if the parameters selected as independent variables met the criteria for statistical significance and if they had an effect on the dependent variable. The *t*-test was employed to perform this type of analysis, and the *p*-value was assumed as statistically significant limit if *p* < 0.05. This means that *p*-values lower than 0.05 denoted that the parameter considered had an effect on the dependent variable. Regression coefficients were also estimated in order to identify the correlation that relates the dependent variable to the independent variables. Statistical analysis was carried out through the program Rstudio applying the linear model (lm).

## 3. Results and Discussion

### 3.1. Morphological Characterization

Cork is a cellular material characterized by closed thin-walled cells. Its alveolar structure is highly symmetric and regular like that of a honeycomb, and it does not display intercellular voids. Cork exhibits three main directions (radial, tangential, and axial) that can be distinguished one from the other thanks to cell shape. Cork closed cells are prismatic with a polygonal base that can vary from four to nine edges. Prisms are stacked base-to-base along the radial direction and this means that, if cork is observed in this direction, a polygonal shape is recognized. Instead, if it is observed in the axial or tangential direction, it displays a brick-layered structure that can be ascribed to the parallel arrangement of cells [6,7,8,17].

**Figure 2:**
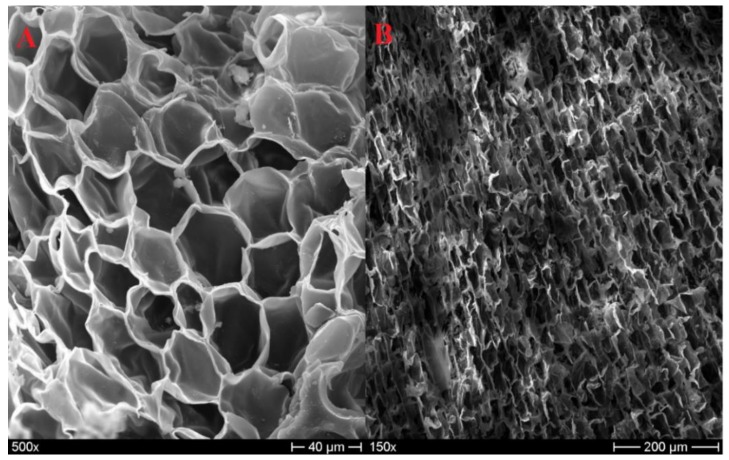
NL20 micrograph in the radial direction (**A**); NL25 micrograph in the tangential/axial direction (**B**).

The morphological analysis conducted in this study on agglomerated cork led to the observation of the same microstructure described above. In Figure 2A, the typical polygonal shape of the prism base can be recognized, whereas, in Figure 2B, the common brick-layered structure of the axial and tangential direction can be observed. Figure 2 also allows detecting the most important feature of cork, i.e., the cell-wall undulation. This characteristic is responsible for the great recovery capabilities of cork. When a compression load is applied, cork cell walls are subjected to buckling, and their corrugation starts to increase. This leads to densification or collapse of the cells without significant damaging or permanent deformation. When the compression load is removed, cell walls are free to unfold, leading to a high dimensional recovery [6].

**Figure 3:**
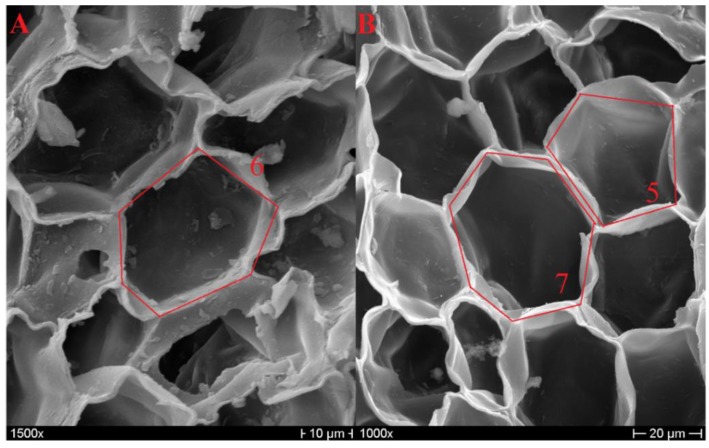
Hexagonal prism base from a NL10 micrograph (**A**); heptagonal and pentagonal base prisms from a NL20 micrograph (**B**).

**Figure 4:**
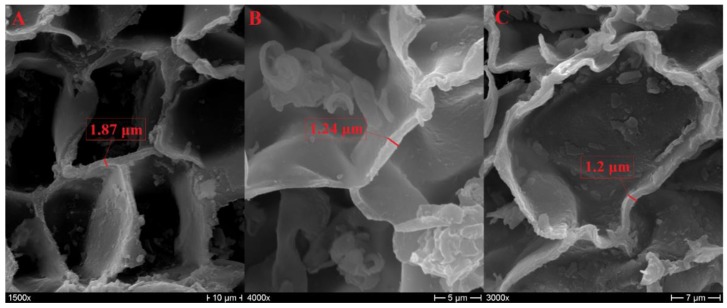
Evaluation of cell-wall thickness of NL10 (**A**), NL20 (**B**), and NL25 (**C**).

As previously mentioned, the number of edges of the polygonal base can vary from four to nine, but the shapes that are most frequent from a statistical point of view are the pentagonal, hexagonal, and heptagonal ones [7,17], as can be observed in Figure 3. In the literature, an average cell-wall thickness of 1–1.5 µm was detected for early cork [6,8], and a thickness up to 2 µm was detected for late cork [7]. Figure 4 reports the measurement of cell-wall thickness of NL10, NL20, and NL25 performed with the program Image J. It is possible to notice that the values obtained are in agreement with those reported in the literature.

**Figure 5:**
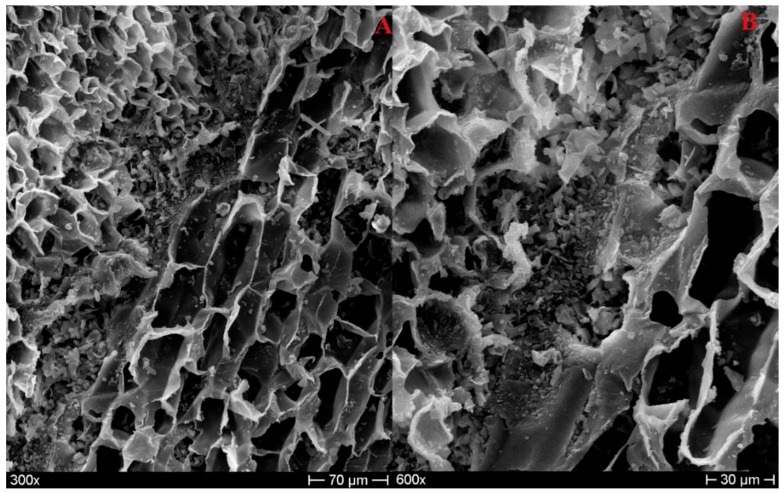
NL10 micrographs of the boundary region between two granules (**A**) with an enlargement of the polymeric binder (**B**).

The cork used in this work was an agglomerated cork, and this means that the different granules were bonded together through a polyurethane binder. In Figure 5, the boundary region between two adjacent granules is depicted. It is possible to observe the presence of the polymeric binder (Figure 5B) and the different orientation of the cells in the two granules.

**Figure 6:**
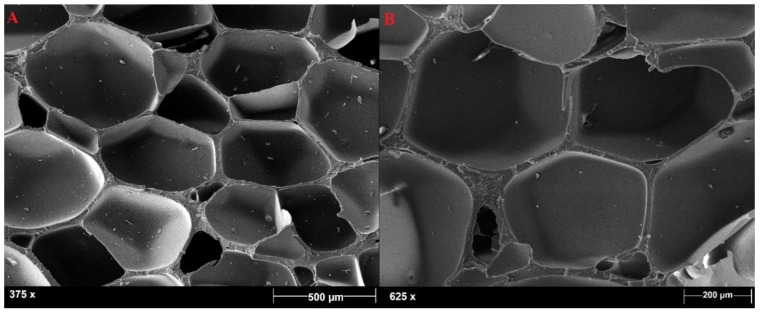
HP250 (**A**) and HP200 (**B**) closed-cell microstructure.

**Figure 7:**
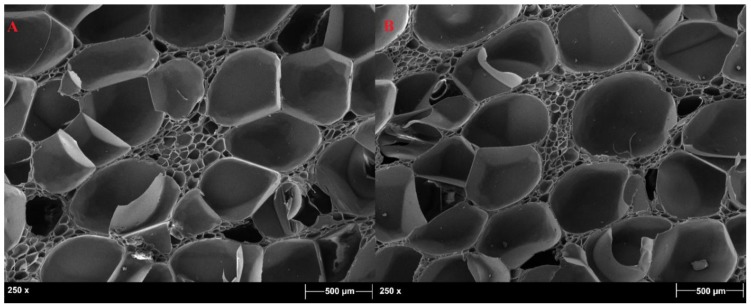
HP130 closed-cell microstructure in two different areas of the sample (**A**) and (**B**).

The PVC foams selected were characterized by a closed-cell microstructure, as shown in Figure 6. This architecture was already observed by Colloca et al. [18] and by Lim and Altstädt [19], who noticed an increase in cell-wall thickness and a decrease in cell size with an increase in foam density. It is noteworthy that HP200 and HP250 were characterized by bulky cell walls that could be easily detected, whereas HP130 was characterized by thinner cell walls and by highly porous zones between the different cells, as can be observed in Figure 7. The main closed cells were surrounded by smaller ones, and this can be ascribed to the fact that HP130 was the foam with the lowest density, and a higher amount of gas had to be inflated in the polymer mass to reach the desired density value.

### 3.2. Surface Wettability

**Figure 8:**
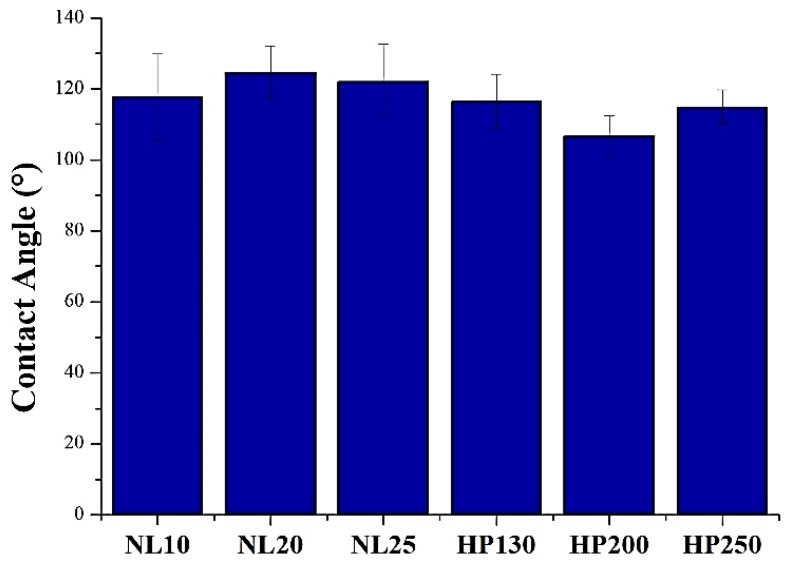
Agglomerated cork and polyvinylchloride (PVC) foam contact angles.

**Figure 9:**
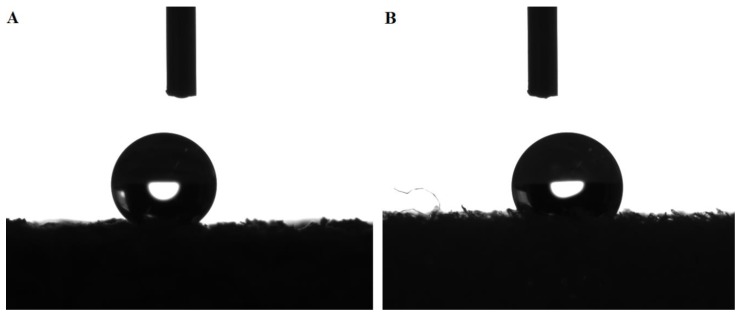
NL20 (**A**) and HP130 (**B**) water droplets.

The average contact angle values are summarized in Figure 8. Agglomerated cork contact angles were between 115° and 125°, whereas PVC foam contact angles varied between 105° and 117°. This means that agglomerated cork displayed a higher hydrophobicity than PVC foam. This conclusion can also be drawn by observing the water droplets of NL20 and HP130 in Figure 9. The hydrophobic nature of cork was already observed by Abenojar et al. [20], who detected a contact angle of natural cork between 90° and 100°. Similar results were obtained by Gomes et al. [21] who observed an average contact angle of 84° ± 2°. These contact angle values are lower than those obtained in this study, and this can be ascribed to the difference between natural cork and agglomerated cork roughness. Agglomerated cork is characterized by the typical roughness of cork and by a macro roughness caused by the discontinuity between granules. It is well known that an increase in roughness tends to reduce the wettability of a hydrophobic material. This hypothesis is also supported by the PVC foam contact angle measurements. The static contact angle of PVC film is usually around 82° [22], which is clearly lower than that obtained in this study. In this case, it must also be considered that the PVC film had a lower roughness than foam.

### 3.3. Thermal Conductivity

**Figure 10:**
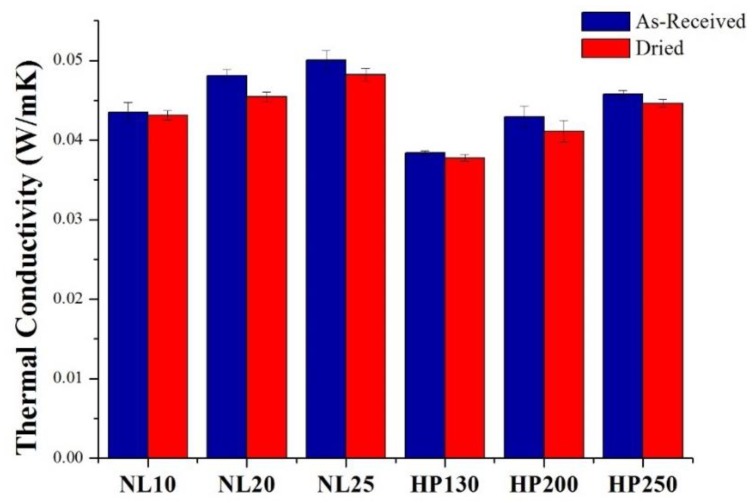
Agglomerated cork and PVC foam thermal conductivity in as-received and dried conditions.

**Figure 11:**
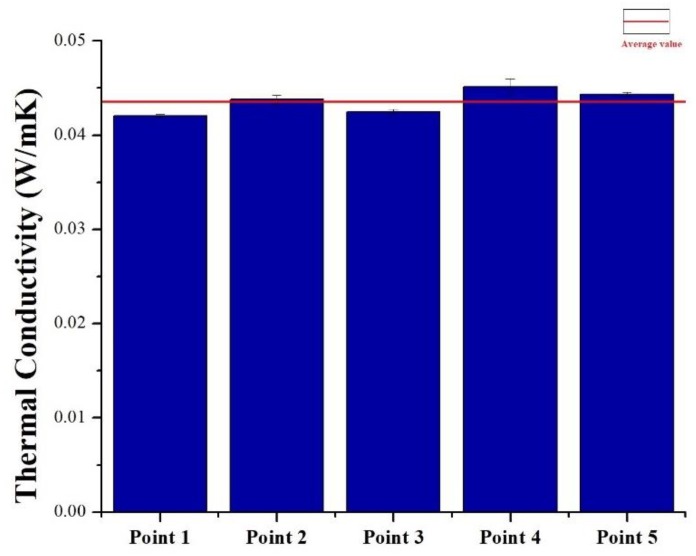
NL10 thermal conductivity evaluated at five different points of the sample.

Thermal conductivity values are summarized in Figure 10. Upon comparing agglomerated cork and PVC foam with the same density, it is possible to notice that cork displayed a higher thermal conductivity for both as-received and dried samples. Both materials displayed a strong dependence of thermal conductivity on density, and, in particular, an increase in density led to an increase in thermal conductivity. This can be explained considering that a decrease in material density was due to an increase in the dry air content that acted as a good thermal insulator. This dependence of thermal conductivity on density can also be noted looking at Figure 11, which represents the average value of NL10 thermal conductivity in the five measurement points of the sample. Considering that the standard deviation of thermal conductivity at each point was very limited, the deviation of the five values of thermal conductivity from the total average value was ascribed to differences in local density that could be met across the sample. In Figure 10, the thermal conductivity dependence on humidity content is also evident. For all materials, a decrease in thermal conductivity was registered for dried samples, and this can be ascribed to the fact that water has a thermal conductivity (0.6 W/mK) higher than cork and PVC by one order of magnitude. Similar results were obtained by Limam et al. [15], who performed their tests on black agglomerated cork taking into account the sample water content, and by Matias et al. [23].

**Figure 12:**
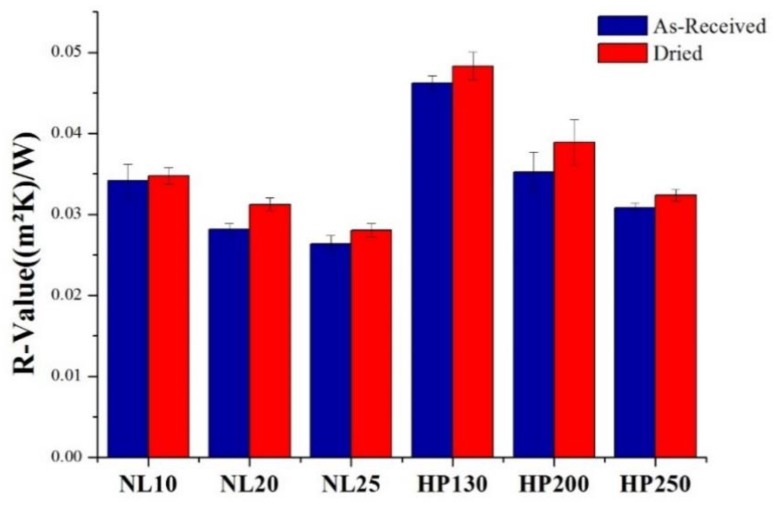
Agglomerated cork and PVC foam *R*-values in as-received and dried conditions.

A parameter that is deeply related to thermal conductivity is the *R*-value or resistance value of insulation, which allows defining how well an insulating material can resist heat flow. The *R*-value is defined as the ratio between material thickness and material thermal conductivity. A higher *R*-value denotes a more effective insulation capability of the material. Considering this and looking at the *R*-value reported in Figure 12, it seems that PVC foams are better insulators than agglomerated cork, and that a lower density led to a higher insulation capability. Even if the *R*-value is an effective parameter, it is not enough to determine how well an insulating material works. The *R*-value takes into account only conduction and does not consider other heat transfer modes such as convection and radiation. Convection and radiation depend on cellular material cell size. Convection does not contribute significantly to heat transfer when the cell size is lower than 1 mm, and this is the case for both PVC foam and agglomerated cork [17]. Radiation instead plays an important role in heat transfer, whereby a smaller cell size gives rise to a longer time necessary to absorb and reradiate heat and a lower rate of flow. Considering that cork cells are much smaller than the cells of any synthetic foam, it displays better insulation properties from a radiation point of view [17].

**Figure 13:**
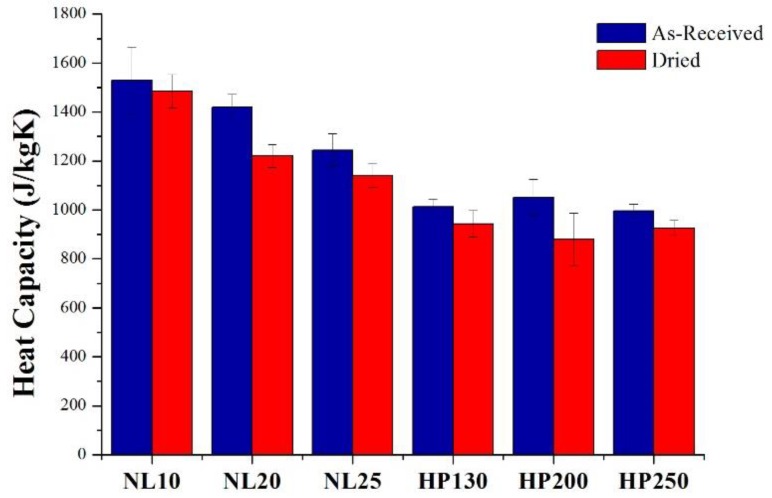
Agglomerated cork and PVC foam heat capacity in as-received and dried conditions.

Heat capacity can be defined as the ability of a material to store heat. Heat capacity values are summarized in Figure 13. Thanks to its honeycomb structure, agglomerated cork is a good heat absorber [15], and it displayed a higher storage capability than PVC foams. The dried sample specific heat was always lower than that of as-received samples, because, even in this case, the water in the liquid (4180 J/kgK) or in the vapor (1996 J/kgK) states had a higher specific heat than cork and PVC.

**Figure 14:**
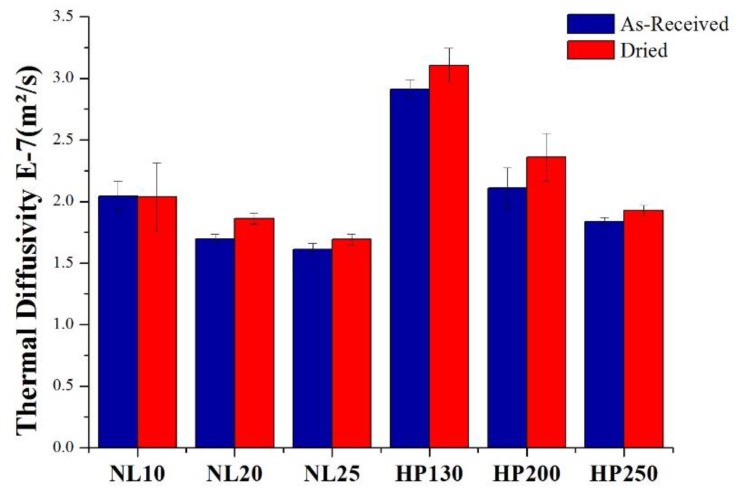
Agglomerated cork and PVC foam thermal diffusivity in as-received and dried conditions.

Thermal diffusivity, a material-specific property for characterizing unsteady heat conduction, is given by the ratio of thermal conductivity and specific heat per unit volume, and it measures the rate of heat transfer through a material. To be more accurate, thermal diffusivity describes how quickly a material reacts to temperature changes. A larger thermal diffusivity results in a faster propagation of heat into the material. In Figure 14, thermal diffusivity values are summarized, and it is possible to note that, even if agglomerated cork displayed a higher thermal conductivity than PVC foams with the same density, it also displayed a lower thermal diffusivity thanks to its major ability to store heat. A lower thermal diffusivity value results in better heat storage by the material [15].

### 3.4. Thermogravimetric Analysis (TGA)

**Figure 15:**
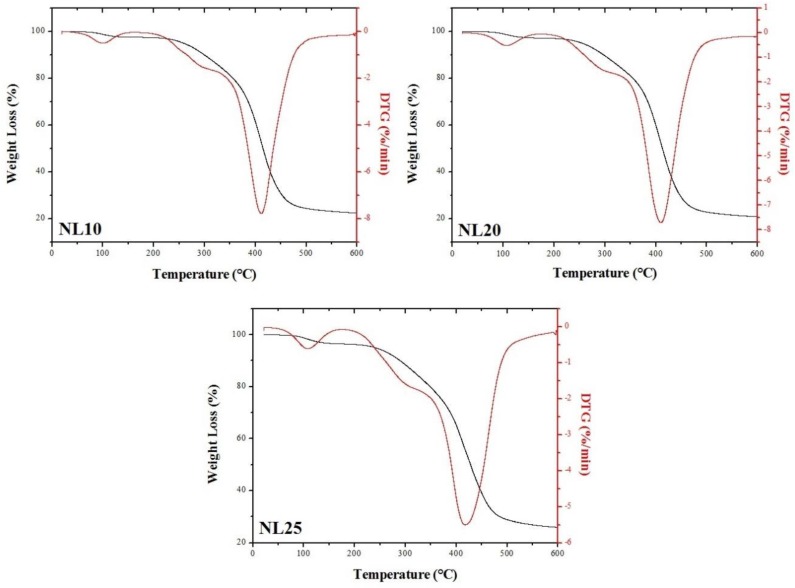
Thermogravimetric analysis (TGA) and DTG curves of NL10, NL20, and NL25.

TGA and DTG curves of NL10, NL20, and NL25 are depicted in Figure 15. At around 100 °C, NL10 displayed a first mass loss (2.6 wt. %) that can be ascribed to the loss of humidity. This temperature shifted to 106 °C and 108 °C in the case of NL20 and NL25, respectively. This temperature increase can be ascribed to an increase in agglomerated cork density, which led to a reduction in porosity that hindered the moisture removal from the sample. Between 200 °C and 330 °C, all three types of agglomerated cork displayed a second mass loss that varied between 11.5 wt. % and 13.5 wt. %. This decomposition step was characterized by two different phenomena. The first one was the thermal decomposition of the polyurethane binder, whereas the second one was the volatilization of extractives and the degradation of hemicellulose. Extractive volatilization was also observed by Sen et al. for natural cork at 315 °C [24], whereas hemicellulose degradation between 220 and 315 °C was observed by Shangguan et al. [25]. A third decomposition step was detected between 410 °C and 418 °C. In this temperature range, suberin and lignin, which are the most stable components, degrade [25]. The final mass loss at 600 °C was 77.5 wt. % for NL10, 79 wt. % for NL20, and 74 wt. % for NL25.

**Figure 16:**
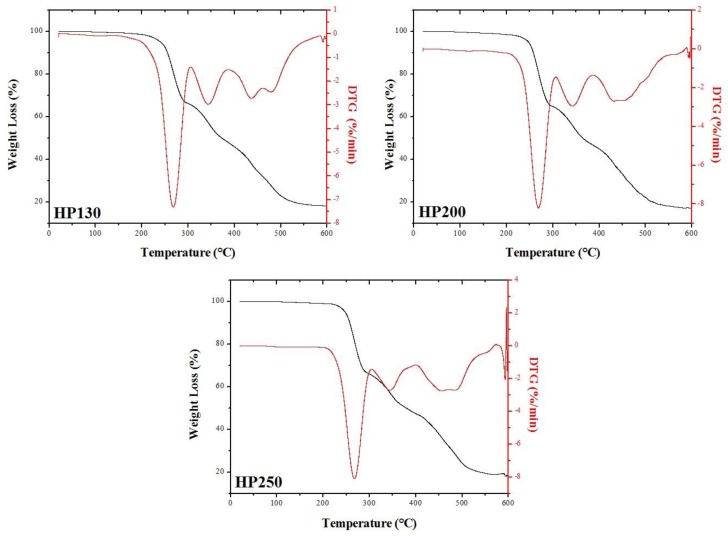
TGA and DTG curves of HP130, HP200, and HP250.

TGA and DTG curves of HP130, HP200, and HP25 are depicted in Figure 16. Even for PVC foams, it was possible to observe a mass loss due to moisture between 110 and 120 °C. At around 268 °C and 343 °C, the PVC foams displayed two degradation steps that led to a huge mass loss (33–36 wt. % for the first step and 18–19 wt. % for the second step). They can be ascribed to the loss of plasticizer and to a polymer dehydrochlorination that caused the emission of HCl, benzene, and vinyl chloride and the formation of a linear polyene structure [26]. A fourth degradation step took place between 430 °C and 490 °C and led to a mass loss between 24.5 wt. % and 27.5 wt. %. In this step, the double bonds of polyene structure were broken, thus leading to the formation of volatile hydrocarbons. The residual mass at 600 °C at the end of the test was in the range from 17 wt. % to 18 wt. %.

### 3.5. Dynamic Mechanical Analysis (DMA)

**Figure 17:**
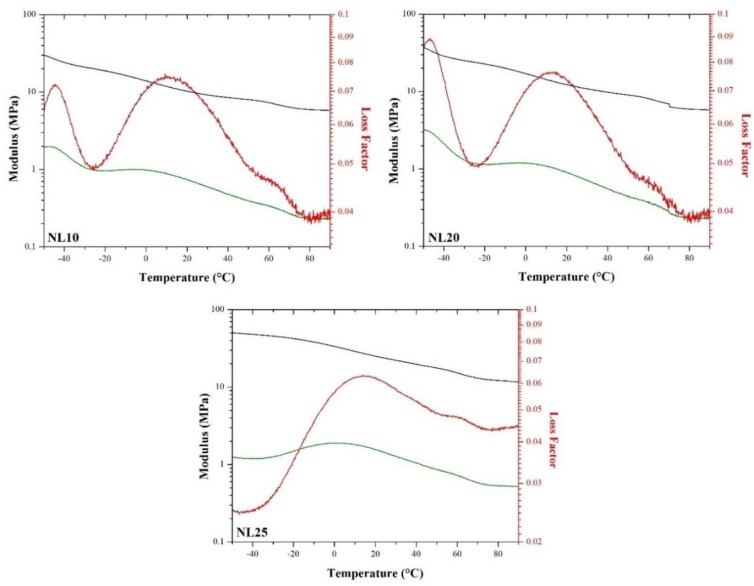
Storage modulus (blue), loss modulus (green), and loss factor (red) of NL10, NL20, and NL25.

**Figure 18:**
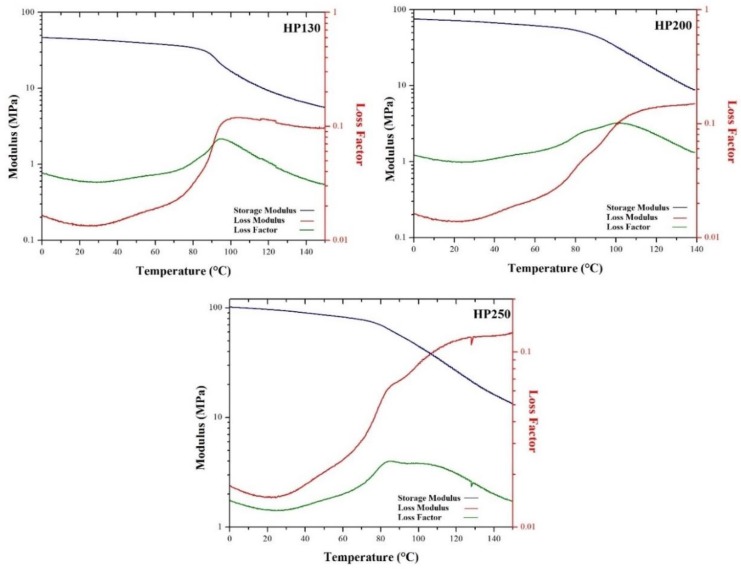
Storage modulus (blue), loss modulus (green), and loss factor (red) of HP130, HP200, and HP250.

DMA curves of agglomerated cork and PVC foams are depicted in Figure 17 and Figure 18, respectively. For both classes of material, it is possible to notice a decrease in storage modulus with rising temperature. With respect to agglomerated cork, the tanδ curves highlighted a double peak. The first one was around −45 °C, ascribed to the glass transition of the polyurethane binder, whereas the second one was strictly related to cork behavior and can be ascribed to suberin melting, as suggested by Paiva and Magalhães [27]. Looking at PVC foam curves, a transition from a glassy state to a viscoelastic state was evident due to the sharp reduction in storage modulus and the sharp increase in loss modulus and tanδ. The same response in storage modulus and loss modulus was detected by Khoshnoud and Abu-Zahra [28]. Table 1 summarizes the glass transition temperature values for both agglomerated cork and PVC foams, which are in perfect agreement with those that can be found in the literature [27,28,29]. It is clear that agglomerated cork works in a viscoelastic state at room temperature, whereas PVC foams work in a glassy state. It is also necessary to underline that agglomerated cork glass transition temperatures are close to room temperature, and this must be taken into account when the material is selected. Possible changes in its mechanical behavior can take place, and this is something that requires further studies.

### 3.6. Shear Test

**Figure 19:**
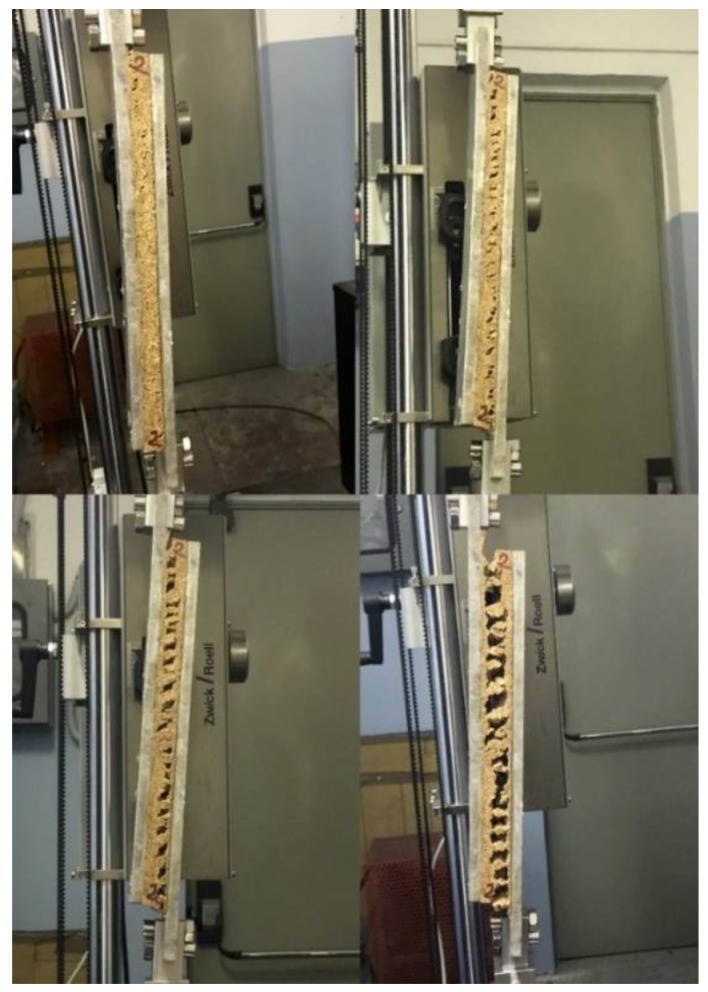
Crack propagation during NL25 shear test.

**Figure 20:**
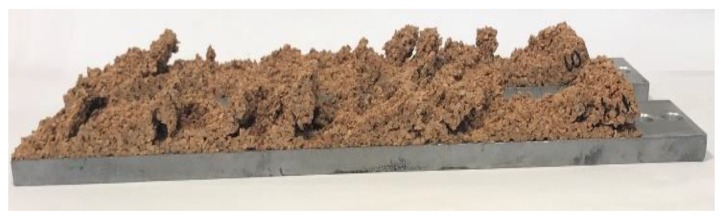
NL10 intergranular fracture after shear test.

Shear modulus and shear strength values are summarized in Table 2. Shear modulus and shear strength increased when cork density increased. After an initial elastic regime, permanent damaging of the sample started to appear in the form of small isolated cracks along the specimen’s diagonal. These little cracks progressively enlarged with the increase of the deformation applied to the sample until they coalesced in a few main cracks, as shown in Figure 19. At this point, the load-bearing capabilities of the sample decreased drastically and final failure occurred. This behavior was previously acknowledged by Reis and Silva [30]. The analysis of cork fracture in shear revealed a fracture of intergranular type for all three types of agglomerated cork, as shown in Figure 20. This type of fracture highlights that sample damaging can be ascribed to binder or binder–cork interface fracture and not to cork granule failure.

### 3.7. Bending Test

**Figure 21:**
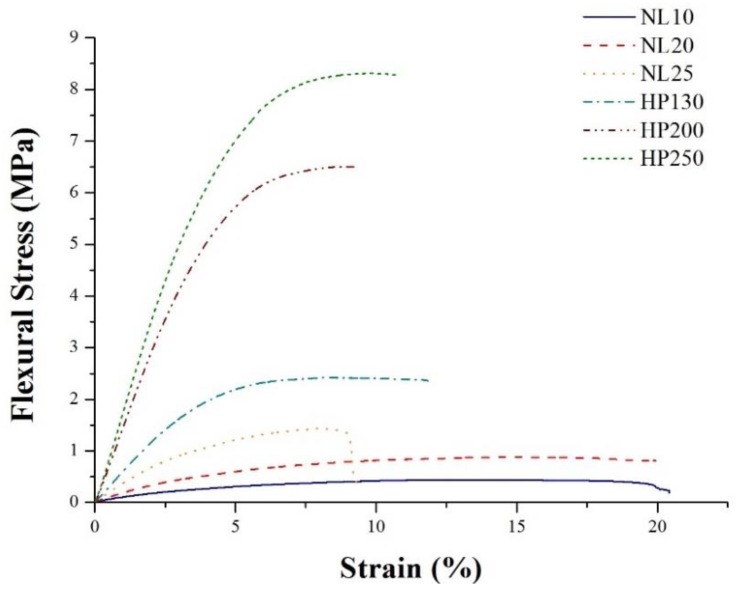
Typical stress—strain curves in bending for core materials at room temperature.

Bending test curves at room temperature are depicted in Figure 21, whereas test results are summarized in Table 3. All PVC foams showed much higher flexural performances than agglomerated cork. Because of the much higher modulus, PVC foams displayed a more brittle behavior than cork, which led to foam fracture at lower deformations. Considering the overall flexural performances, the foam that displayed the closest behavior to agglomerated cork was HP130, i.e., the foam with the lowest density.

**Figure 22:**
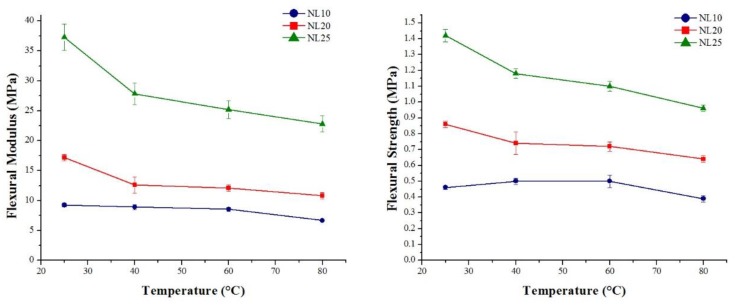
Flexural modulus and strength as a function of temperature for agglomerated corks.

**Figure 23:**
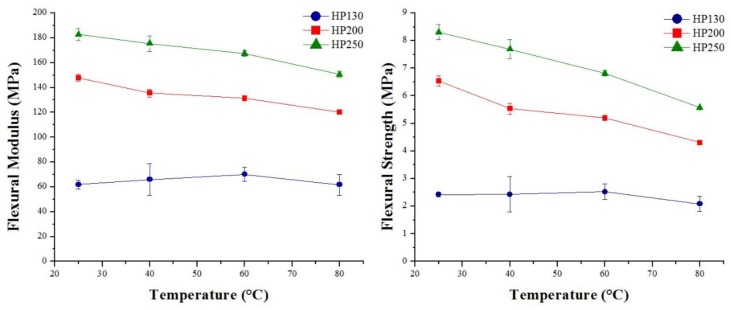
Flexural modulus and strength as a function of temperature for PVC foams.

**Figure 24:**
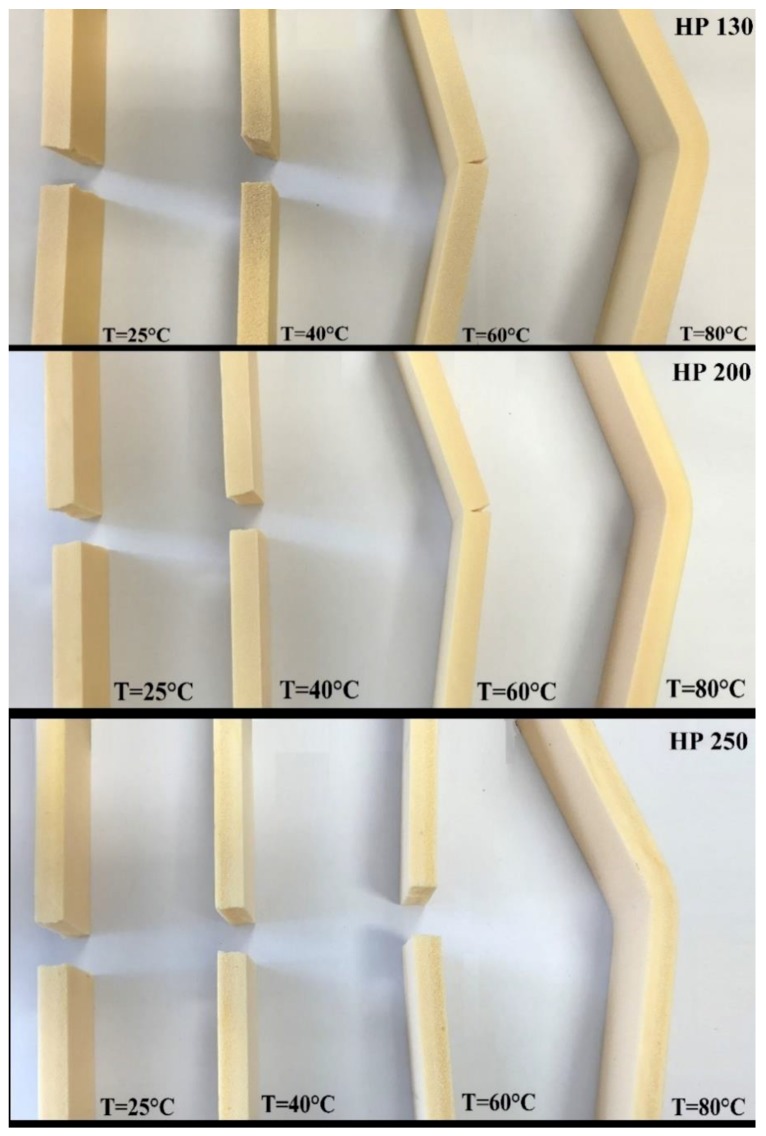
Failure modes in bending for PVC foams as a function of temperature.

Figure 22 and Figure 23 summarize the flexural modulus and the strength values of the three families of agglomerated cork and PVC foams as a function of temperature. For all materials, it is possible to observe that an increase in temperature led to a progressive decrease in both flexural modulus and strength. The behavior of flexural modulus is perfectly consistent with the results obtained by DMA. Considering that storage modulus is an index of the material’s capability to store energy in an elastic way, its continuous decrease with temperature allowed explaining the decrease in flexural modulus. With respect to PVC foams, the main drop in flexural modulus can be observed at 80 °C. This result can be explained through the help of DMA, which highlighted a glass transition temperature between 77 °C and 85 °C. This statement is also confirmed by the PVC foam damage mode depicted in Figure 24, where all PVC foams displayed a brittle fracture until 60 °C, whereas they did not show any type of damage when tested at 80 °C. With regard to the reduction of agglomerated cork flexural strength, this can be ascribed to a progressive degradation of binder mechanical performance with temperature.

Data were statistically analyzed to confirm the results, and, from the data gathered in Table 4 and Table 5, it is clear that density (*ρ*), temperature (*T*), and their interaction (*ρT*) displayed statistical significance, denoting that they actually have an effect on the mechanical properties. The statistical significance was so high that, in almost all cases, the chances of the results being due to error alone were less than 1%, which is a significance limit lower than that usually chosen, i.e., 5% (*p* < 0.05). The estimation of the regression coefficients of each parameter led to the following relationships (Equations (1)–(4)):

*E*_PVC_ = −78.57 + 1.13 *ρ*_PVC_ + 0.61 *T*_PVC_ − 0.005 *ρ*_PVC_*T*_PVC_, (1)

*σ*_max___PVC_ = −4.75 + 0.058 *ρ*_PVC_ + 0.04 *T*_PVC_ − 3.64 × 10^−4^*ρ*_PVC_*T*_PVC_, (2)

*E*_CORK_ = −29.56 + 0.27 *ρ*_CORK_ + 0.22 *T*_CORK_ − 0.0017 *ρ*_CORK_*T*_CORK_, (3)

**Figure 25:**
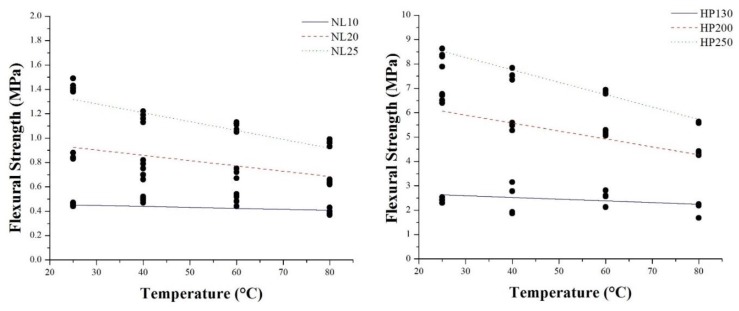
Flexural strength dataset of agglomerated corks and PVC foams, and fitting curves with constant density in the three cases.

*σ*_max___CORK_ = −0.84 + 9.35 × 10^−3^*ρ*_CORK_ + 7.44 × 10^−3^*T*_CORK_ − 5.89 × 10^−5^*ρ*_CORK_*T*_CORK_, (4)

where Equation (1) allows evaluating the PVC foam flexural modulus (*E*), Equation (2) allows evaluating the PVC foam flexural strength (*σ*_max_), Equation (3) allows evaluating the agglomerated cork flexural modulus, and Equation (4) allows evaluating the agglomerated cork flexural strength. The correlation between experimental datasets and the linear model was very accurate, as shown by the coefficients of determination summarized in Table 6. The good correlation between experimental data and the fitting curves can be also noted by observing the graphs in Figure 25.

### 3.8. Compression Test

**Figure 26:**
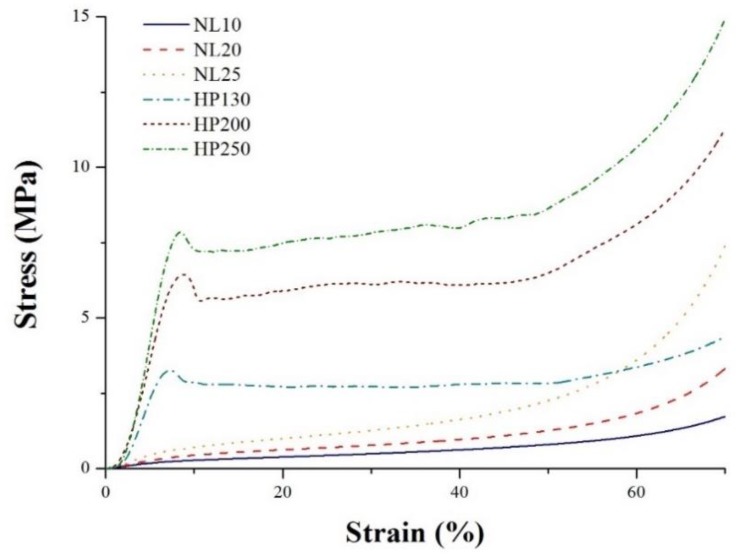
Typical compression curves for PVC foams and agglomerated corks.

Typical compression curves are depicted in Figure 26 for all materials. PVC foams exhibited a higher elastic modulus and a higher plateau stress than all agglomerated cork types. A higher foam density led to a higher elastic modulus and yield strength. This can be explained by taking into account that a higher density can be ascribed to thicker cell walls, as previously mentioned in the morphological characterization, which are more resistant against buckling [18,19]. An increase in elastic modulus and plateau stress with density can also be observed for agglomerated cork. These results are in agreement with those obtained by Anjos et al. [12,31] who studied cork compressive behavior as a function of density and test direction. A great difference in PVC foam and agglomerated cork behavior in terms of compression can also be noted. Synthetic foam curves were characterized by four main regions. It was possible to identify an initial elastic region controlled by the cell-wall elastic deformation that went up to the yielding point where material plastic deformation and cell collapse started. The third region was the plateau region where cell buckling and collapse occurred until the fourth region, where densification took place [18,19]. Agglomerated cork displayed a quite different behavior characterized by three main phases that were extensively studied by Anjos et al. [12,31], Rosa and Fortes [13], and Pereira [6]. The first region was an elastic region where the deformation was ascribed to cell-wall elastic bending. The elastic regime extended up to approximately 5% and was followed by a wide plateau region. This plateau extended from 5% to 60–70% strain, identifying the buckling of the cells. The final strain value of this region was strictly related to cork density; in fact, the third part of the curve was the densification region that appeared earlier when cork density was higher [32]. In the densification phase, cell walls start touching each other and experience mutual contact. It is important to highlight that compression does not cause cell-wall fracture or failure thanks to the suberin macromolecule, which allows cell-wall undulation and complete folding [6]. When the compressive load is removed, cork is able to almost recover its original dimensions thanks to the unfolding of cell walls. The limited permanent deformation can be ascribed to pores and lenticular channel deformation.

**Figure 27:**
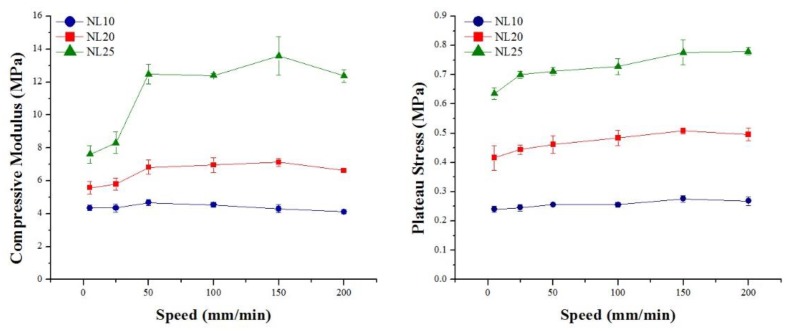
Agglomerated cork compressive modulus and plateau stress as a function of test speed.

**Figure 28:**
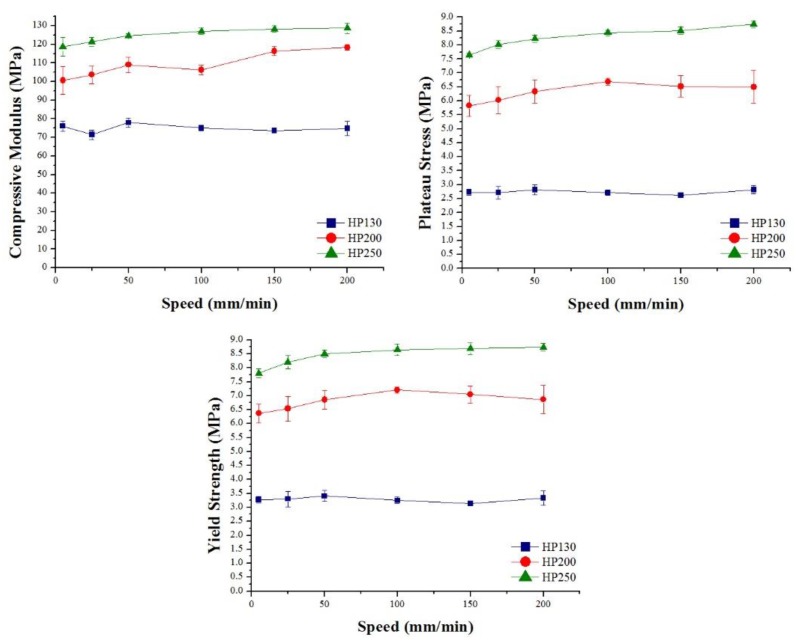
PVC foam compressive modulus, plateau stress, and yield strength as a function of test speed.

Core material strain rate sensitivity was investigated, and the results obtained are summarized in Figure 27 and Table 7 for agglomerated cork, and in Figure 28 and Table 8 for the PVC foams. With regard to agglomerated cork, an increase in compressive modulus and plateau stress was detected, and these results are in agreement with those obtained by Rosa and Fortes [13], who studied the strain rate sensitivity of cork planks in the three main directions, and with those obtained by Le Barbenchon et al. [33], who studied the strain rate sensitivity of an agglomerated cork produced with a thermoset resin reinforced with short fibers. The increase in plateau stress can be explained by taking into account that, when test speed increased, the material struggled to adapt to the deformation, thus leading to a higher reaction force. The highest variation in compressive properties was detected for NL25, which displayed a change of 43% in the elastic modulus and a change of 18% in plateau stress. This can be explained considering that a higher density results in a higher amount of material that has to adapt to deformation. With respect to PVC foams, strain rate sensitivity analysis displayed a more complex scenario. In agreement with the results obtained by Daniel et al. [34], the PVC compressive modulus did not display a dependence on test speed, whereas a little increase in yield strength and plateau stress could be observed, especially for the two foams with higher density. The results obtained by Daniel et al. displayed more clearly the dependence of the two parameters on test speed because the speed working range was broader, whereas, in this study, the tests were all performed in quasi-static conditions.

In this case, the results obtained were also supported by statistical analysis. In Table 9 and Table 10, the statistical significance of the effect of density (*ρ*), speed (*v*), and their interaction (*ρv*) on compressive modulus (*E*) and plateau stress (*σ*_plateau_) of agglomerated cork and PVC foams is summarized. With respect to agglomerated cork, the effect of test speed on both compressive modulus and plateau stress was confirmed by its statistical significance. For PVC foams, statistical analysis provided good support for the experimental data; in fact, no statistical significance was detected for the test speed effect on PVC compressive modulus, in perfect agreement with the experimental results. With respect to PVC plateau stress, statistical significance was detected for the test speed effect. Its value was lower than the selected limit of *p* < 0.05, but it was higher than the value related to density, which was lower than *p* < 0.01. This can be explained taking into account, as mentioned previously, that even if test speed had an effect on PVC foam yield strength and plateau stress, the speed working range was not broad enough to detect it markedly. The estimation of the regression coefficients of each parameter led to Equations (5)–(7).

*E*_CORK_ = −1.285 + 0.039 *ρ*_CORK_ − 0.037 *v*_CORK_ + 2.37 × 10^−4^*ρ*_CORK_*v*_CORK_, (5)

*σ*_plateau___CORK_ = −0.3023 + 3.81 × 10^−3^*ρ*_CORK_ − 4.76 × 10^−4^*v*_CORK_ + 4.49 × 10^−6^*ρ*_CORK_*v*_CORK_, (6)

*σ*_plateau___PVC_ = −2.75 + 0.043 *ρ*_PVC_ − 5.17 × 10^−3^*v*_PVC_ + 4.08 × 10^−5^*ρv*_PVC_, (7)

where Equation (5) allows evaluating the agglomerated cork compressive modulus, Equation (6) allows evaluating the agglomerated cork plateau stress, and Equation (7) allows evaluating the PVC plateau stress. The correlation between the datasets and the linear model was very accurate, as can be inferred from the coefficients of determination summarized in Table 11.

**Figure 29:**
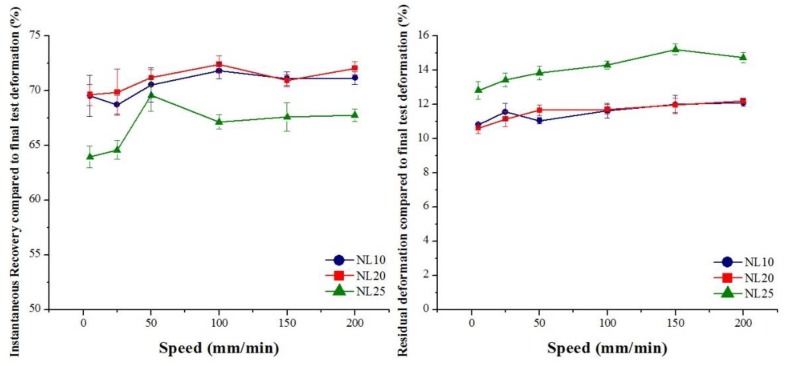
Agglomerated cork instantaneous recovery compared to final test deformation, and residual deformation compared to final test deformation as a function of test speed.

**Figure 30:**
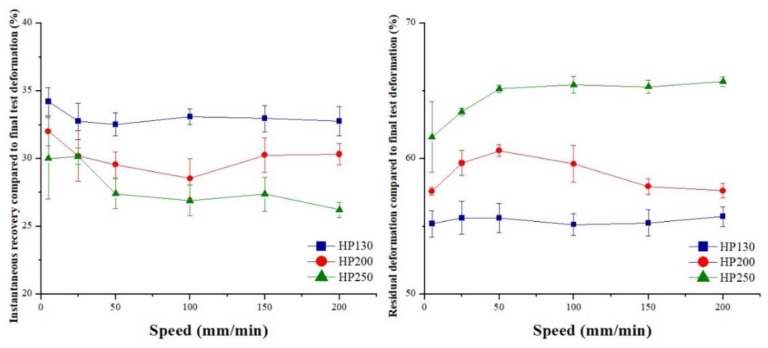
PVC foam instantaneous recovery compared to final test deformation, and residual deformation compared to final test deformation as a function of test speed.

Core material recovery capabilities were investigated, and the results obtained are shown in Figure 29 for agglomerated cork and in Figure 30 for PVC foam. First of all, it is important to highlight that the instantaneous dimension recovery of cork was between 65% and 70%, whereas that of PVC foams was between 25% and 35%, which was mainly due to elastic recovery. With respect to the residual deformation, agglomerated cork displayed values between 10% and 15%, whereas PVC foam values were between 55% and 65%. This remarkable difference in behavior can be explained by taking into account the different material morphology. The rigid cell walls of PVC foams undergo fracture and collapse when submitted to a compressive load and prevent any recovery of the initial dimension. The corrugation of cork cell walls allows the compressive deformation to undergo complete folding without undergoing fracture. When the compressive load is removed, cell walls are free to unfold, ensuring an extensive recovery of dimensions [6]. From the data depicted in Figure 29, it is possible to note that an increase in agglomerated cork density led to a lower instantaneous recovery and to a higher residual deformation for every test speed. This can be ascribed to the fact that a higher cork density resulted in a lower number of cells per unit area [31]. Considering that recovery capabilities are due to cell-wall unfolding, a higher number of cells per unit area leads to higher recovery capabilities of the material. From the dataset, it is also evident that an increase in test speed led to an increase in instantaneous recovery and to an increase in residual deformation for all types of agglomerated cork. The increase in instantaneous recovery can be explained by considering that, when the load is applied faster, the material has a lower reaction time and struggles to deform. This leads to a higher disorder in cell-wall folding, and this suggests that cell walls recover their deformation faster when the load is removed. The increase in residual deformation can be ascribed to the fact that higher disorder in cell folding increases the risk of pore and lenticular channel deformation and collapse, which are the main mechanisms responsible for cork permanent deformation.

**Figure 31:**
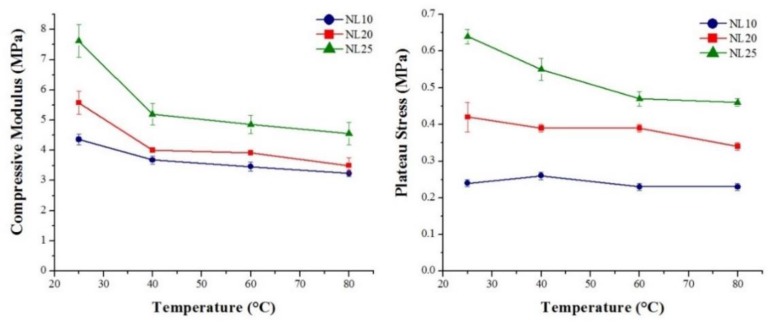
Agglomerated cork compressive modulus and plateau stress as a function of temperature.

**Figure 32:**
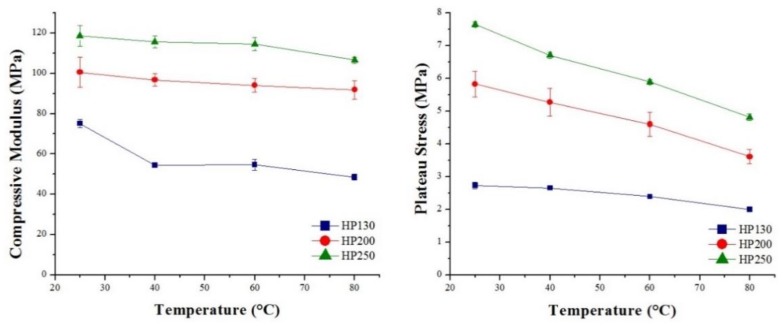
PVC foam compressive modulus and plateau stress as a function of temperature.

The temperature influence on compressive performance was also investigated; in Figure 31 and Figure 32, the effect of temperature on the compressive modulus and plateau stress of agglomerated cork and PVC foams is shown. The compressive performances displayed by both materials were comparable with the trend of flexural performance, thus implying that the same mechanisms were involved.

## 4. Conclusions

In this paper, an overview of agglomerated cork properties was provided, together with a comparison with more traditional PVC foams. The results obtained highlighted a strict correlation between material morphology and mechanical properties. Thanks to cell-wall undulation, agglomerated cork experiences high deformation in compression without undergoing fracture or damaging, with a concurrent recovery capability due to cell-wall unfolding. This characteristic is one of the main advantages of cork over PVC foams, which undergo cell-wall collapse when high compression deformations are reached. In view of all these issues, even if agglomerated cork displays lower quasi-static properties than PVC foams with comparable densities, its recovery capabilities are of great interest in all the fields where energy absorption and damage tolerance after multiple impacts are required. A deeper understanding of mechanical properties as a function of temperature was possible thanks to the support of DMA analysis, which allowed identifying the transition regions of the material from the glassy state to the viscoelastic state. A strong correlation between thermal properties and morphology was also observed. Even if PVC foams display a lower thermal conductivity than agglomerated cork, the insulating capabilities of the material are also dependent on other contributions to heat transfer, i.e., radiation and convection. The smaller dimension of cork cells, for example, allows reducing the radiation effect. The thermal diffusivity of agglomerated cork was found to be lower than that of PVC foams, thus resulting in a slower heat propagation throughout the material.

## Figures and Tables

**Table 1:** Glass transition temperature evaluated by dynamic mechanical analysis (DMA).

Sample	Glass Transition Temperature (°C)
NL10	11.75 ± 0.78
NL20	10.90 ± 2.12
NL25	15.80 ± 1.27
HP130	85.40 ± 0.56
HP200	79.80 ± 1.98
HP250	76.80 ± 0.99

**Table 2:** Agglomerated cork shear modulus and shear strength.

Sample	Shear Modulus (MPa)	Shear Strength (MPa)
**NL10**	1 ± 0.05	0.26 ± 0.04
**NL20**	1.26 ± 0.04	0.57 ± 0.03
**NL25**	1.92 ± 0.27	0.75 ± 0.04

**Table 3:** Core material flexural modulus and strength at room temperature.

Samples	Flexural Modulus (MPa)	Flexural Strength (MPa)
NL10	9.24 ± 0.32	0.46 ± 0.01
NL20	17.20 ± 0.52	0.86 ± 0.02
NL25	37.28 ± 2.20	1.42 ± 0.04
HP130	62.00 ± 3.49	2.42 ± 0.08
HP200	147.80 ± 2.68	6.54 ± 0.19
HP250	182.80 ± 4.71	8.31 ± 0.27

**Table 4:** Statistical significance of the effect of density, temperature, and their interaction on agglomerated cork and polyvinylchloride (PVC) foam flexural modulus.

Flexural Modulus Parameter	Agglomerated Cork Pr (>|t|)	PVC Foam Pr (>|t|)
Density	4.67 × 10^−14^	<2.00 × 10^−16^
Temperature	3.05 × 10^−2^	4.17 × 10^−3^
Density × Temperature	6.08 × 10^−4^	2.10 × 10^−5^

**Table 5:** Statistical significance of the effect of density, temperature, and their interaction on agglomerated cork and PVC foam flexural strength.

Flexural Strength Parameter	Agglomerated Cork Pr (>|t|)	PVC Foam Pr (>|t|)
Density	<2.00 × 10^−16^	<2.00 × 10^−16^
Temperature	4.42 × 10^−4^	7.09 × 10^−5^
Density × Temperature	1.71 × 10^−7^	5.22 × 10^−10^

**Table 6:** Multiple *R*^2^ values for all interpolation models.

Dependent Variable	Multiple *R*^2^
PVC foam flexural modulus	0.976
PVC foam flexural strength	0.978
Agglomerated cork flexural modulus	0.865
Agglomerated cork flexural strength	0.951

**Table 7:** Agglomerated cork compressive modulus and plateau stress values as a function of test speed.

Test Speed (mm/min)	Compressive Modulus (MPa)			Plateau Stress (MPa)		
	NL10	NL20	NL25	NL10	NL20	NL25
5	4.36 ± 0.18	5.58 ± 0.38	7.62 ± 0.54	0.24 ± 0.01	0.42 ± 0.04	0.64 ± 0.02
25	4.36 ± 0.25	5.80 ± 0.38	8.32 ± 0.66	0.25 ± 0.01	0.44 ± 0.02	0.70 ± 0.01
50	4.68 ± 0.19	6.82 ± 0.43	12.48 ± 0.61	0.26 ± 0.01	0.46 ± 0.03	0.71 ± 0.01
100	4.54 ± 0.15	6.96 ± 0.44	12.40 ± 0.12	0.26 ± 0.01	0.48 ± 0.03	0.73 ± 0.03
150	4.30 ± 0.25	7.12 ± 0.25	13.60 ± 1.17	0.28 ± 0.01	0.51 ± 0.01	0.78 ± 0.04
200	4.12 ± 0.13	6.62 ± 0.11	12.38 ± 0.40	0.27 ± 0.01	0.50 ± 0.02	0.78 ± 0.01

**Table 8:** PVC foam compressive modulus and plateau stress values as a function of test speed.

Test Speed (mm/min)	Compressive Modulus (MPa)			Plateau Stress (MPa)		
	HP130	HP200	HP250	HP130	HP200	HP250
5	75.08 ± 2.13	100.58 ± 7.43	118.68 ± 5.15	2.73 ± 0.10	5.82 ± 0.38	7.65 ± 0.08
25	71.52 ± 2.54	103.57 ± 4.61	121.40 ± 2.35	2.71 ± 0.23	6.02 ± 0.49	8.02 ± 0.14
50	67.92 ± 2.24	109 ± 4.10	124.62 ± 1.14	2.82 ± 0.18	6.34 ± 0.42	8.2 ± 0.13
100	65.78 ± 0.63	106.20 ± 2.77	127 ± 1.69	2.71 ± 0.10	6.68 ± 0.13	8.44 ± 0.10
150	66.56 ± 2.87	116.36 ± 2.47	128.2 ± 1.83	2.61 ± 0.04	6.52 ± 0.38	8.51 ± 0.14
200	70.68 ± 4.74	118.30 ± 1.46	128.68 ± 2.89	2.82 ± 0.15	6.50 ± 0.58	8.75 ± 0.13

**Table 9:** Statistical significance of the effect of density, test speed, and their interaction on agglomerated cork and PVC foam compressive modulus.

Compressive Modulus Parameter	Agglomerated Cork Pr (>|t|)	PVC Foam Pr (>|t|)
Density	3.33 × 10^−12^	<2.00 × 10^−16^
Speed	6.09 × 10^−5^	9.15 × 10^−2^
Density × Speed	4.18 × 10^−7^	-

**Table 10:** Statistical significance of the effect of density, test speed, and their interaction on agglomerated cork and PVC foam compressive plateau stress.

Plateau Stress Parameter	Agglomerated Cork Pr (>|t|)	PVC Foam Pr (>|t|)
Density	<2.00 × 10^−16^	<2.20 × 10^−16^
Speed	2.91 × 10^−2^	1.27 × 10^−2^
Density × Speed	6.01 × 10^−5^	1.27 × 10^−4^

**Table 11:** Multiple *R*^2^ values for all interpolation models.

Dependent Variable	Multiple *R*^2^
Agglomerated cork compressive modulus	0.842
Agglomerated cork plateau stress	0.975
PVC foam plateau stress	0.981
